# Supplementary material for: Deforestation amplifies climate change effects on warming and cloud level rise in African montane forests
Source: Nat Commun. 2024 Aug 14;15:6992. doi: 10.1038/s41467-024-51324-7 (PMC11324879; doi:10.1038/s41467-024-51324-7)
Supplement: Supplementary file 1 — Supplementary Information [file 41467_2024_51324_MOESM1_ESM.pdf]

Supplementary information for

Deforestation amplifies climate change effects on warming and cloud level rise in African montane forests

Temesgen Alemayehu Abera[1,2*], Janne Heiskanen[2,3], Eduardo Eiji Maeda[2,3], Mohammed Ahmed Muhammed[1,4], Netra Bhandari[1], Ville Vakkari[3,5], Binyam Tesfaw Hailu[2,4], Petri K.E. Pellikka[2,6], Andreas Hemp[7], Pieter G. van Zyl[5], and Dirk Zeuss[1]

[1]Department of Environmental Informatics, Faculty of Geography, Philipps-Universität Marburg, Deutschhausstraße 12, 35037 Marburg, Germany
[2]Department of Geosciences and Geography, University of Helsinki, P.O. Box 68, FI-00014, Helsinki, Finland
[3]Finnish Meteorological Institute, P.O. Box 503, FI-00101 Helsinki, Finland
[4]School of Earth Sciences, Addis Ababa University, Addis Ababa, Ethiopia
[5]Atmospheric Chemistry Research Group, Chemical Resource Beneficiation, North-West University, Potchefstroom, South Africa
[6]State Key Laboratory for Information Engineering in Surveying, Mapping and Remote Sensing, Wuhan University, Wuhan 430079, China
[7]Department of Plant Systematics, University of Bayreuth, 95440, Bayreuth, Germany

Correspondence to: temesgen.abera@geo.uni-marburg.de

The following figures and tables are included in this pdf:

Supplementary Figure 1: Deforestation-induced dewpoint temperature change map
Supplementary Figure 2: Seasonality of deforestation-induced T_{max} and cloud base height change
Supplementary Figure 3: Contribution of climate change and deforestation to air temperature change
Supplementary Figure 4: Deforestation-induced cloud water interception efficiency loss map
Supplementary Figure 5: Reference samples locations for evaluating accuracy of forest loss estimate
Supplementary Figure 6: Spatial prediction map of T_{max}, T_{min}, and T_{dew}
Supplementary Figure 7: Location map of the GSOD weather station data used for model training and validation
Supplementary Figure 8: Scatterplot comparing precited vs measured T_{max} for each month
Supplementary Figure 9: Scatterplot comparing precited vs measured T_{min} for each month
Supplementary Figure 10: Scatterplot comparing precited vs measured T_{dew} for each month
Supplementary Figure 11: Spatial cross-validation test for T_{max}, T_{min}, and T_{dew} model
Supplementary Figure 12: Validation site description in Bale Mountain in Ethiopia
Supplementary Figure 13: Validation site description in Vuria Mountain in Kenya
Supplementary Figure 14: Validation site description in Kilimanjaro Mountain in Tanzania
Supplementary Figure 15: Independent validation of T_{max}, and T_{min} against measured data
Supplementary Figure 16: Comparison of modeled and measured (Ceilometer) CBH data in South Africa
Supplementary Figure 17: Comparison of T_{air} and T_{dew} from ERA5-Land and MERRA-2 against in situ data
Supplementary Table 1: Summary of model performance statistics for T_{max}
Supplementary Table 2: Summary of model performance statistics for T_{min}
Supplementary Table 3: Summary of model performance statistics for T_{dew}
Supplementary References: 1–13

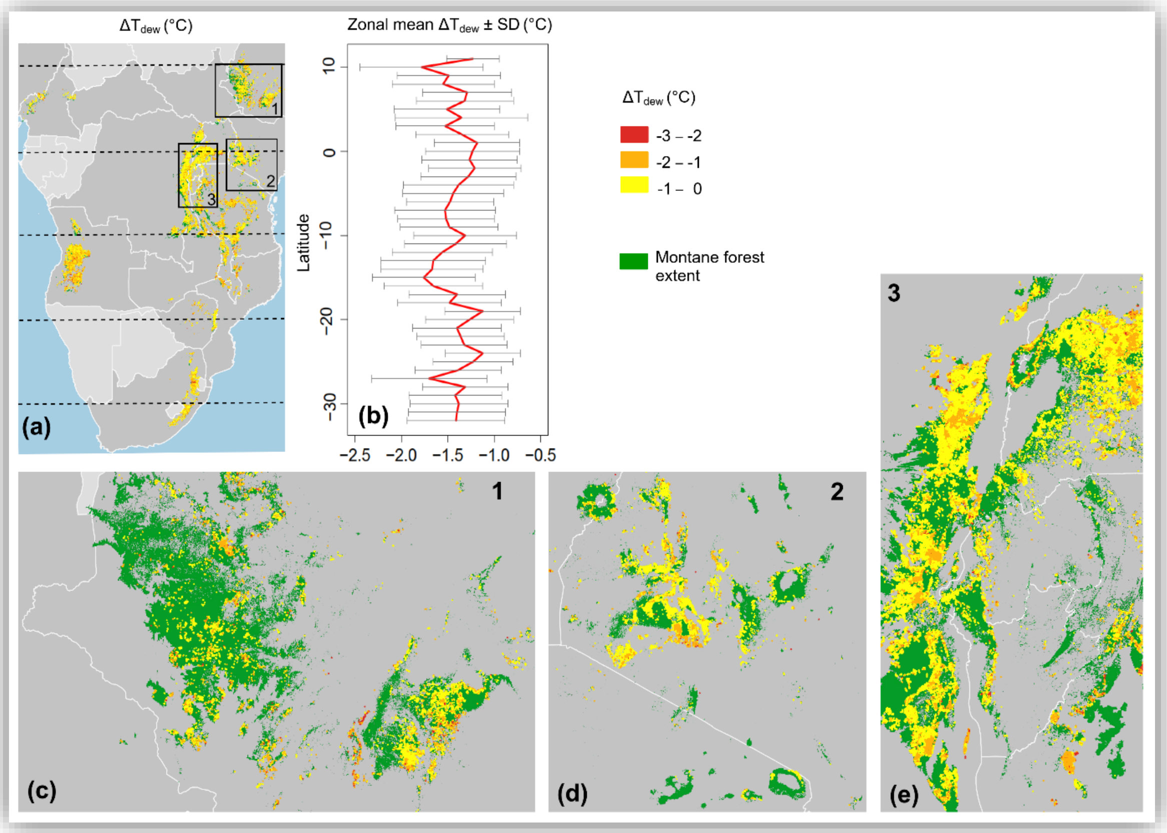

Supplementary Figure 1. Panel (a) shows deforestation-induced dewpoint temperature change (ΔT_{dew}) across montane forest in Africa. Panel (b) shows deforestation-induced mean ΔT_{dew} and standard deviation (SD) across 2° latitude interval. Closer view of ΔT_{dew} over Ethiopia (c), Kenya (d), and Central to Western part of eastern Africa (e) is displayed for boxes (1–3) in Panel (b). Montane forest cover extent (see Methods in the main manuscript) is used as a background. Administrative boundary data are from Global Administrative Areas (GADM) (2015 - 2022).

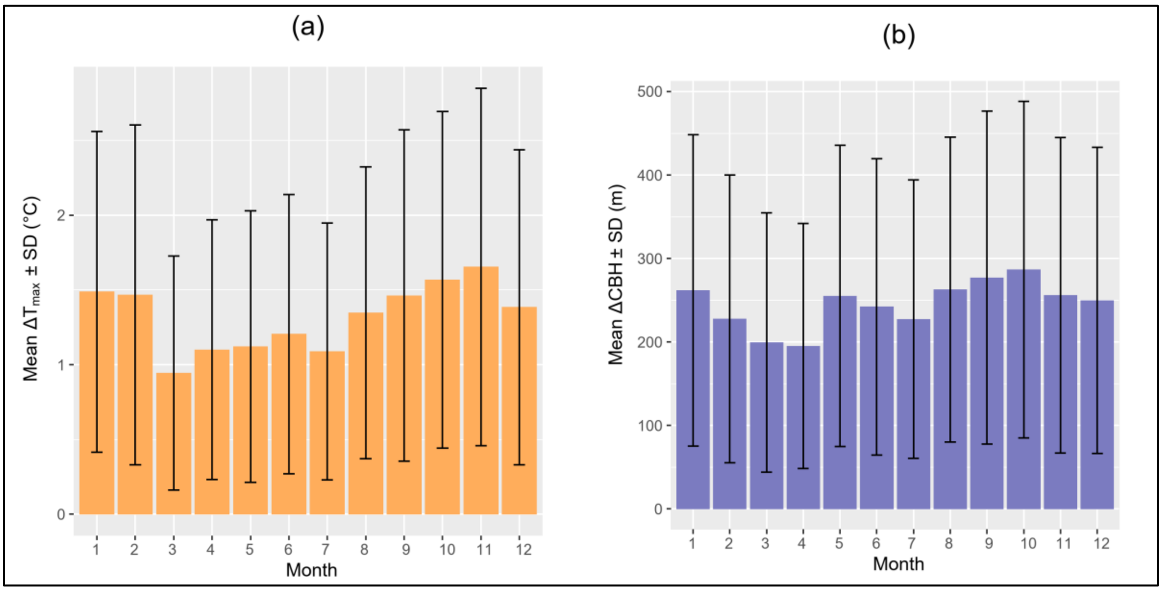

Supplementary Figure 2. Seasonality of deforestation-induced (a) T_{max} and (b) CBH change in montane forest in Africa. The error bar shows mean ± standard deviation (SD).

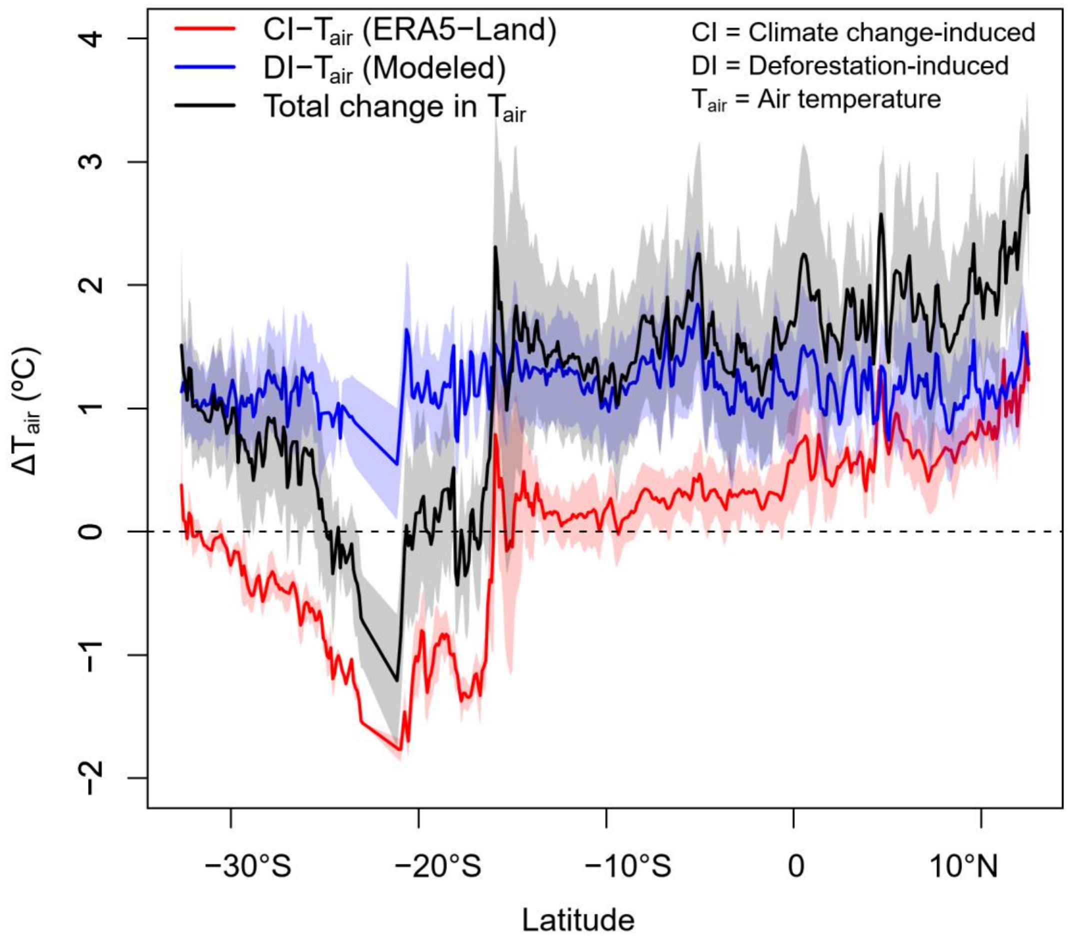

Supplementary Figure 3: Contribution of climate change and deforestation to maximum air temperature (ΔT_{air}) change in tropical montane forest of Africa across latitude from South to North at ~ 10 km interval. For climate change-induced ΔT_{air}, 30 years of ERA5-land T_{air} data between 1992 to 2022 were used. Deforestation-induced changes were between 2003 and 2022. The blue, red, and black line show the average ΔT_{air} due to deforestation, climate change, and combined effect from these two factors. The shaded region show mean ± standard deviation.

Estimating cloud water interception efficiency loss due to deforestation

We computed the spatial distribution of cloud water interception efficiency (CWI) loss using Katata et al. (2008) method, which was developed for montane cloud forest. The CWI, which refers to the deposition velocity of cloud water, depends on the vegetation structure (Leaf area index and canopy height) and can be estimated using the below equation. The equation was reported to have good correlation (R^2=0.928) against numerical experiment (Katata et al., 2008).

$$CWI = 0.0164[LAD \times (m)]^{-0.5}$$

$$LAD = \frac{LAI}{H}$$

$$\Delta CWI = \frac{\Delta LAI}{\Delta H}$$

where LAD= leaf area density in m2/ m3; LAI= leaf area index (m2/ m2); H= canopy height (m). We used H data at 30 m spatial resolution for the year 2000 and 2020 from Potapov et al. (2022). The H data was prepared by integratig Landsat analysis-ready data, which is valid for time series analysis, and Global Ecosystem Dynamics Investigation (GEDI) Lidar based on machine learning ensemble approach (Potapov et al., 2021 & 2022). For the corresponding forest loss pixels indetified in the main manuscript during 2003 - 2022, we calculated ΔH= H (2000) - H (2020). Since our forest loss anaysis excluded pixels with tree cover losses occured before 2003, the H (2000) is applicabe to H (2003). However, there could be underestimation in ΔCWI due to the two years difference between H (2020) and the forest loss period (i.e., 2003 - 2022). For leaf area index (LAI), we used the 2003 average LAI from Moderate Resolution Imaging Spectroradiometer (MODIS) MCD15A3H Version 6.1 product, which is a 4-day composite product combined from Aqua and Terra sensor at 500 m resolution (Myneni et al., 2021). We assumed near zero (0.1) LAI after forest loss (i.e., in 2022) to reduce uncertainty arising from the LAI product (Pu et al. 2020).

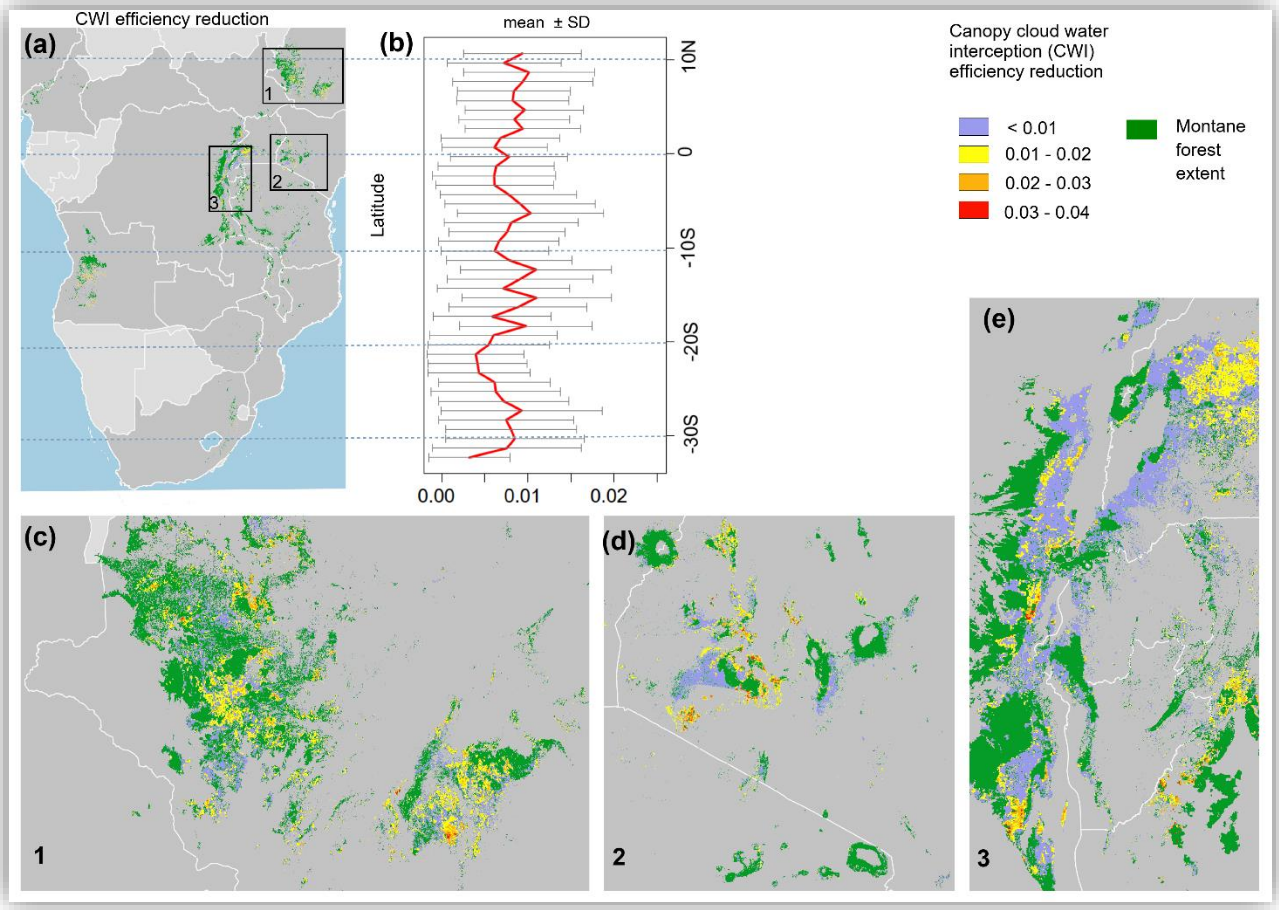

Supplementary Figure 4. Panel (a) shows deforestation-induced cloud water interception efficiency (CWI) reduction across montane forest in Africa. Higher value shows stronger reduction in CWI. Montane forest extent (see Methods in the main manuscript) is used as a background. Panel (b) shows the deforestation-induced mean ΔCWI and standard deviation (SD) across 1° latitude intervals. Closer views of ΔCWI over Ethiopia (c), Kenya (d), and central to western parts of eastern Africa (e) are displayed for boxes (1–3) in Panel (b). Administrative boundary data are from Global Administrative Areas (GADM) (2015 - 2022).

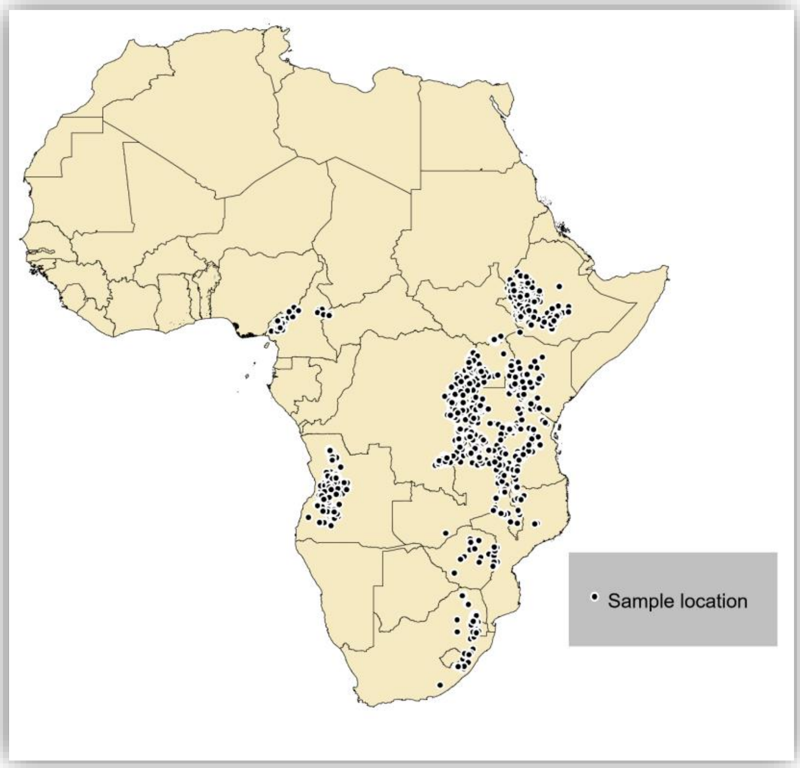

Supplementary Figure 5. Location of samples (1416 samples in total) used for evaluating the accuracy and area uncertainty of the forest loss estimates applying Olofsson et al. 2014 approach. Reference classification (i.e., forest loss and stable class) was done manually using high-resolution Google imagery and Planet monthly mosaics in Google Earth Engine (GEE) (see details of the methodology in our previous study in Abera et al., 2023). Administrative boundary data are from Global Administrative Areas (GADM) (2015 - 2022).

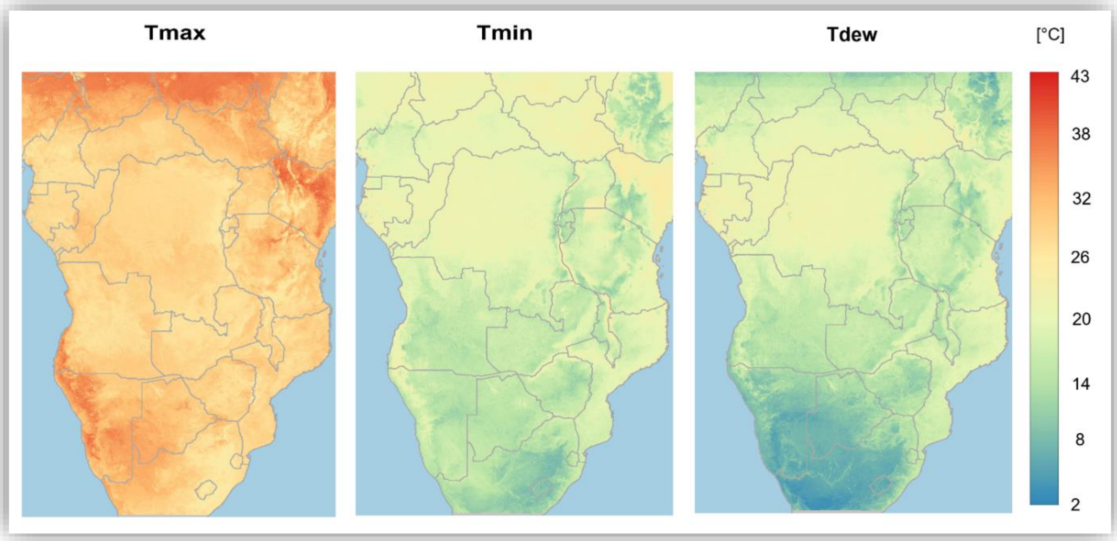

Supplementary Figure 6. The annual average maximum temperature (Tmax), minimum temperature (Tmin), and dew-point temperature (Tdew) predicted using ensemble learning approach in Africa for the year 2003. Administrative boundary data are from Global Administrative Areas (GADM) (2015 - 2022).

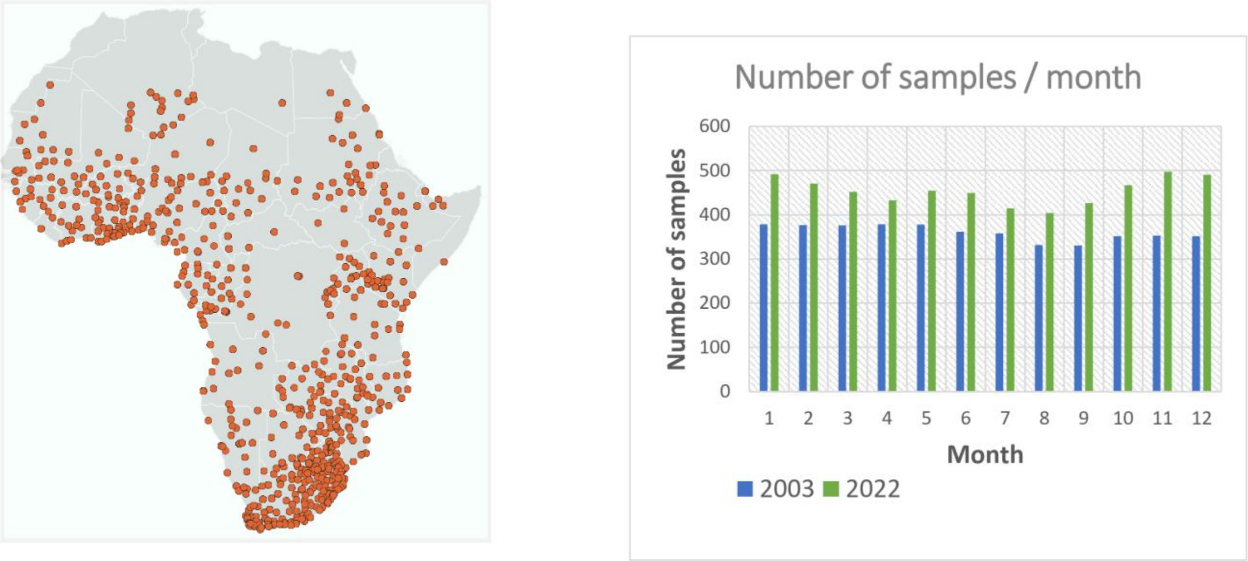

Supplementary Figure 7. Location of the Global Summary of the Day (GSOD) weather station data used for model training and validation. Data are obtained from the National Climate Data Center, (NOAA National Centers of Environmental Information ,1999). The bar plot shows the number of samples for each month in 2003 and 2022. Administrative boundary data are from Global Administrative Areas (GADM) (2015 - 2022).

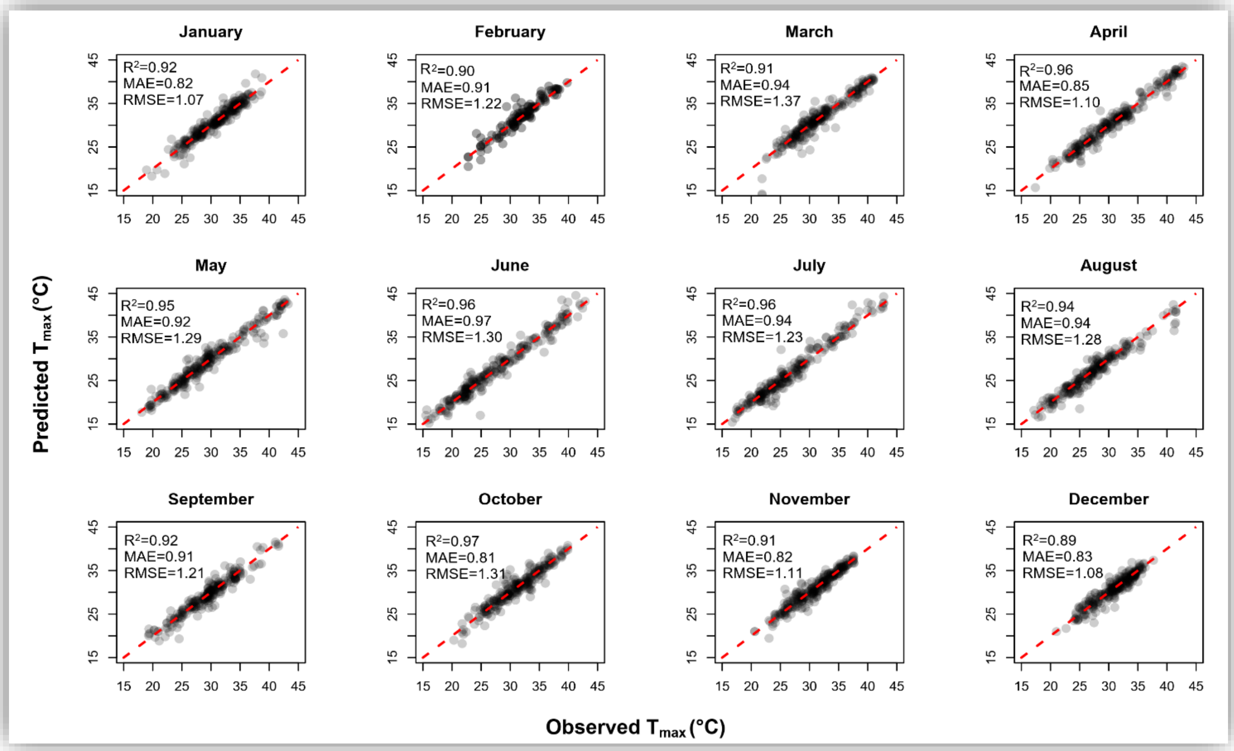

Supplementary Figure 8. Comparison of predicted and observed monthly maximum air-temperature (T_{max}) for each month between 2003 and 2022 in montane forest in Africa. Model training and validation data is from the global summary of the day (GSOD) station data over Africa (NOAA National Centers of Environmental Information ,1999).

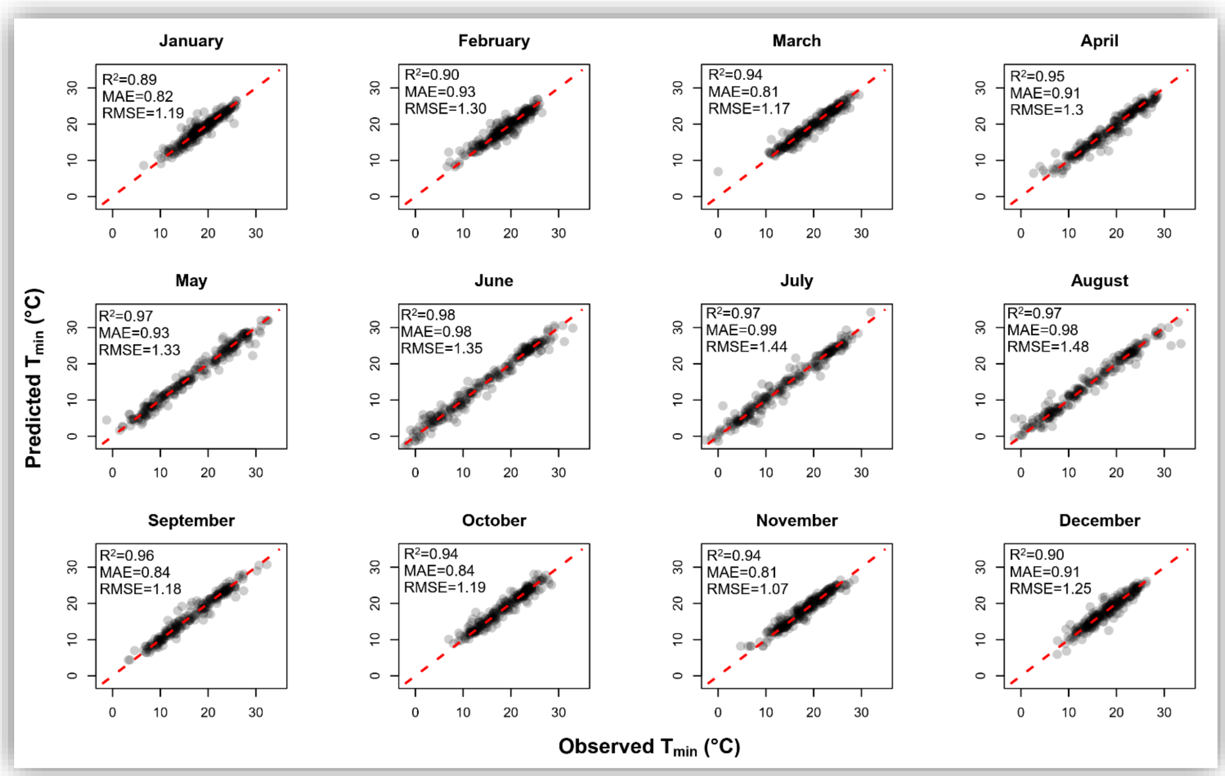

Supplementary Figure 9. Comparison of predicted and observed monthly minimum air temperature (T_{min}) for each month between 2003 and 2022 in montane forest in Africa. Model training and validation data is from the global summary of the day (GSOD) station data over Africa (NOAA National Centers of Environmental Information ,1999).

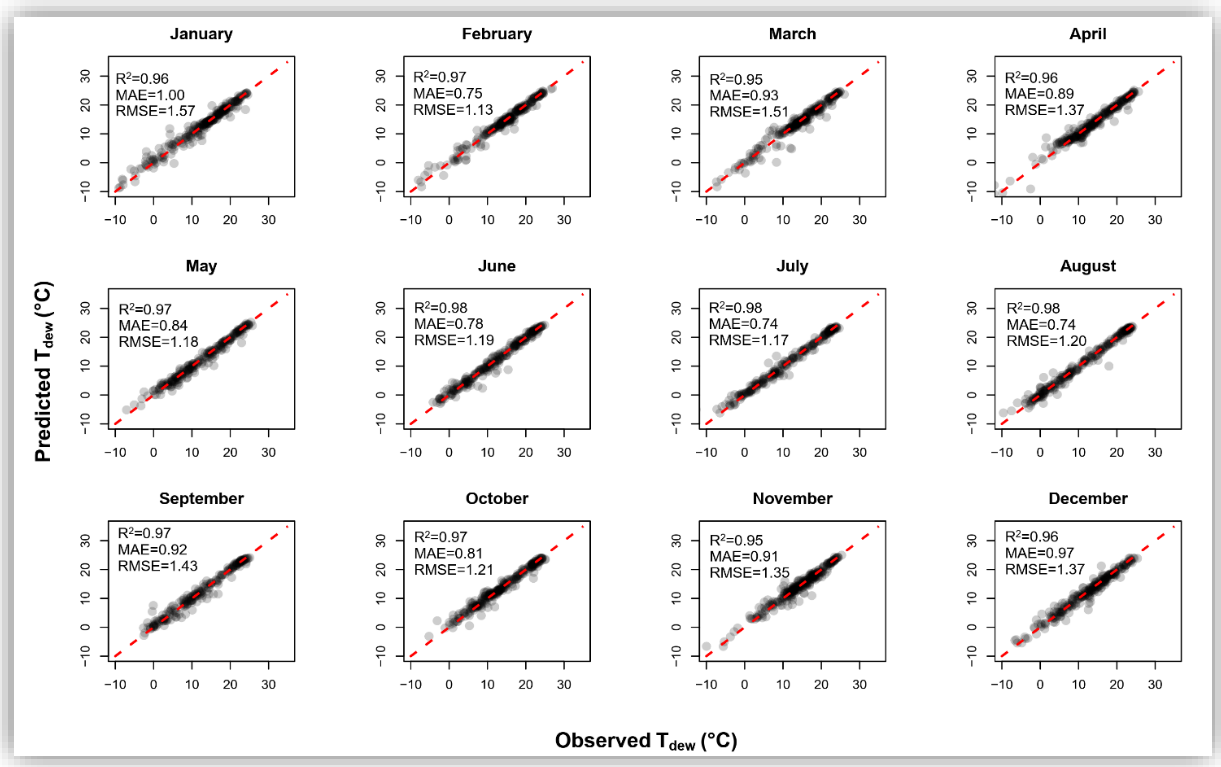

Supplementary Figure 10. Comparison of predicted and observed monthly dew point temperature (T_{dew}) for each month between 2003 and 2022 in montane forest in Africa. Model training and validation data is from the global summary of the day (GSOD) station data over Africa (NOAA National Centers of Environmental Information ,1999).

Table 1. Model performance tested against validation data for T_{max} using R^2, RMSE, and MAE.

	Month											
	1	2	3	4	5	6	7	8	9	10	11	12
R^2	0.920	0.900	0.912	0.965	0.956	0.962	0.959	0.939	0.909	0.900	0.913	0.887
RMSE	0.828	0.916	0.945	0.853	0.922	0.971	0.937	0.943	0.922	0.970	0.820	0.828
MAE	1.067	1.218	1.368	1.104	1.292	1.305	1.232	1.282	1.208	1.312	1.111	1.082

Table 2. Model performance tested against validation data for T_{min} using R^2, RMSE, and MAE.

	Month											
	1	2	3	4	5	6	7	8	9	10	11	12
R^2	0.897	0.901	0.936	0.953	0.973	0.977	0.970	0.968	0.962	0.941	0.942	0.903
RMSE	1.187	1.304	1.176	1.300	1.331	1.356	1.442	1.481	1.185	1.188	1.068	1.254
MAE	0.820	0.936	0.812	0.915	0.932	0.981	0.993	0.982	0.839	0.841	0.811	0.906

Table 3. Model performance tested against validation data for T_{dew} using R^2, RMSE, and MAE.

	Month											
	1	2	3	4	5	6	7	8	9	10	11	12
R^2	0.959	0.974	0.954	0.96	0.975	0.977	0.983	0.982	0.968	0.969	0.951	0.962
RMSE	1.572	1.133	1.513	1.374	1.181	1.192	1.169	1.199	1.430	1.209	1.348	1.375
MAE	1.001	0.747	0.931	0.887	0.841	0.785	0.746	0.746	0.917	0.813	0.907	0.967

Miscellaneous information related to temperature model

The random forest regression models for $T_{max\ (Terra)}$ and $T_{max\ (Aqua)}$ were run using the following parameter settings: number of trees = 500, mtry = 2, and nodesize = 5. The two models were ensembled to generate the final T_{max} model using random forest as meta-learner applying the default parameter setting. For $T_{min(Terra)}$ and $_{Tmin(Aqua)}$ model, similar parameter settings to that of T_{max} were used. The variable importance, in a decreasing order, were: $LST_{day(10:30\ am)}$, longitude, latitude, NDVI, and albedo for $T_{max\ (Terra)}$ model; $LST_{day(1:30\ pm)}$, longitude, latitude, NDVI, and albedo for $T_{max\ (Aqua)}$ model; $LST_{night(10:30\ pm)}$, longitude, latitude, albedo, and NDVI for $LST_{min(Terra)}$ model; $LST_{night(1:30\ am),}$ longitude, latitude, albedo, and NDVI for $LST_{min(Aqua)}$ model.

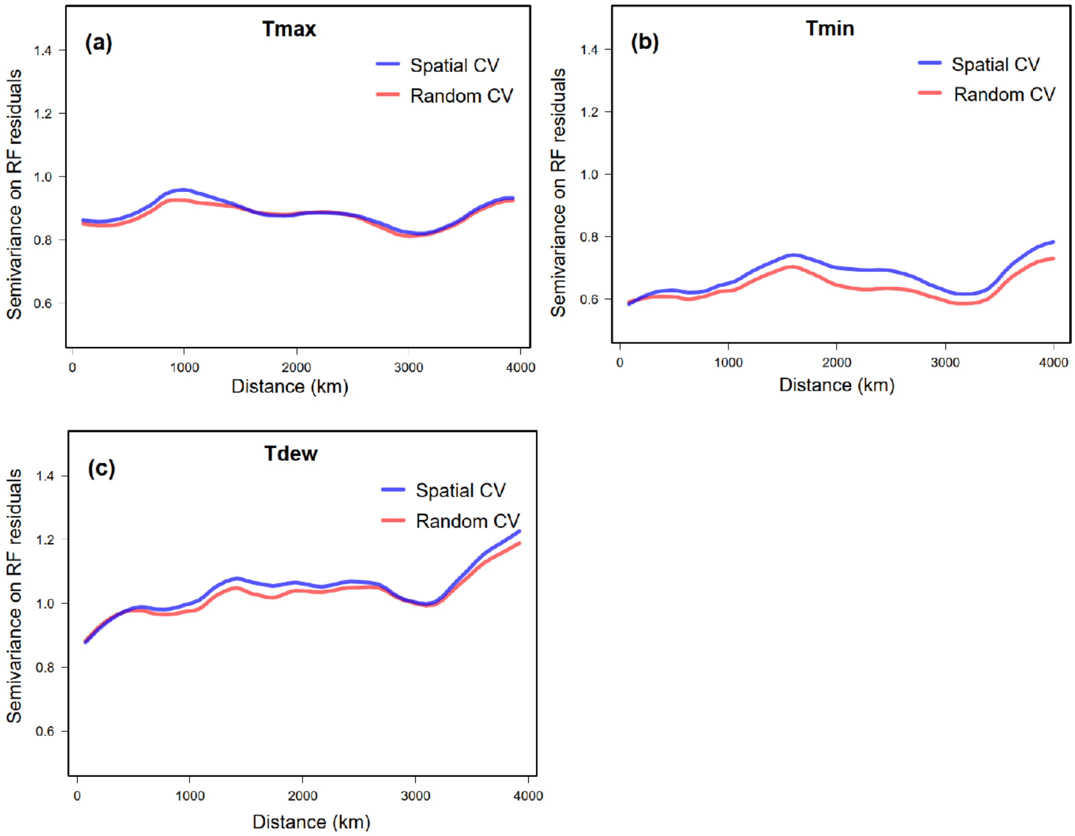

Supplementary Figure 11. Semivariogram showing spatial autocorrelation of (a) maximum air temperature (T_{max}), (b) minimum air temperature (T_{min}), and (c) dewpoint air temperature (T_{dew}). Random forest (RF) model residuals were computed using 10-fold random cross-validation and 68-fold spatial cross-validation (Ploton et al., 2020).

Site description

Bale, Ethiopia

The automatic weather stations in Bale Mountain are located (6.421 – 6.527° N, 39.746 – 39.834° E) in the lower montane forest in the central highlands of Ethiopia with elevation ranging between 1300 and 1530 m a.s.l (Figure 12).The meteorological data were collected every 15-minutes and aggregated hourly.

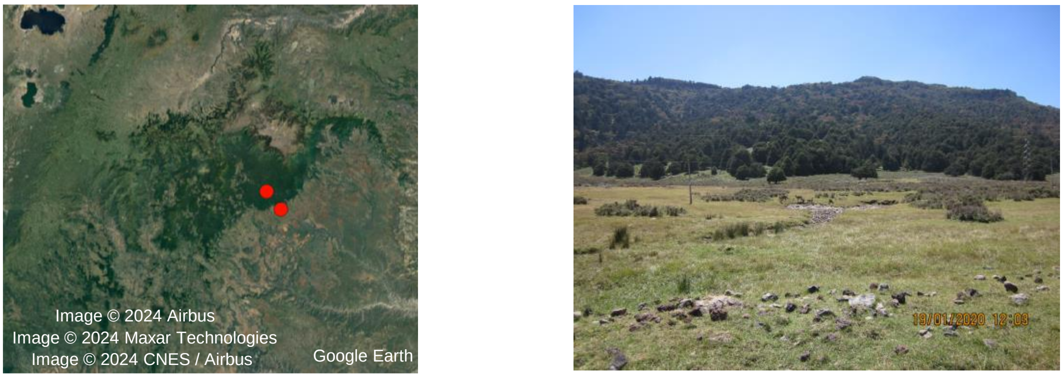

Supplementary Figure 12. Locations of weather stations used for external validation in Bale montane forest in Ethiopia. The background imagery in the left is from Google Earth (sources: Airbus, Maxar Technologies, and CNES). Photo in the right, Mohammed Muhammed ©2020.

Vuria, Kenya

Vuria automatic weather station is located (3.41388889 °S, 38.29138889 °E) in an opening in Vuria montane forest at 2176 m a.s.l in Taita Hills in Kenya (Figure 13). Meteorological data (including air temperature and relative humidity) were sampled every minute and recorded every hour from October 2013 to February 2016 (Räsänen et al., 2018). The dewpoint temperatures were calculated from the relative humidity.

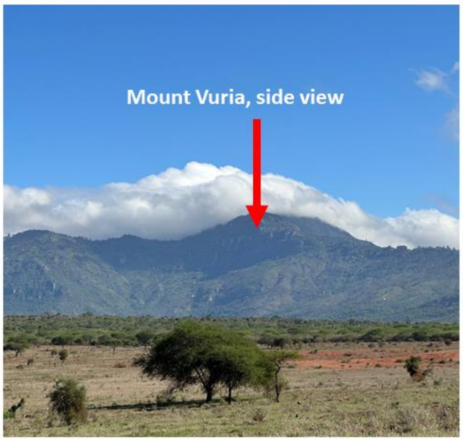

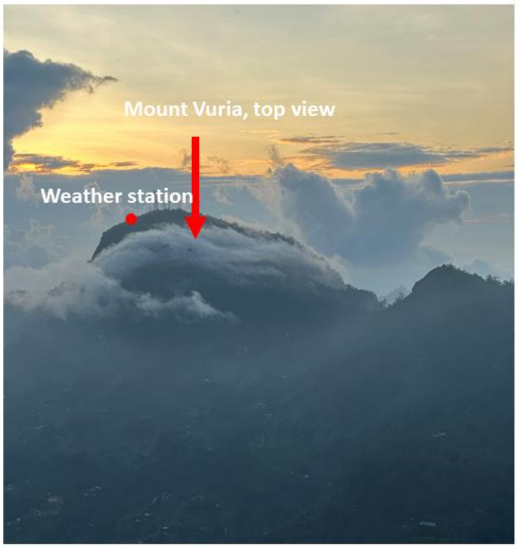

Supplementary Figure 13. Photo of Vuria cloud mountain forest in Kenya from side and top view. Location of weather station is indicated in red dot. Photo, Prof. Petri Pellikka ©2024.

Kilimanjaro, Tanzania

The weather stations are located in the southern Kilimanjaro montane forest (Figure 14). For measuring the local climate, the ambient air temperature and relative humidity (TRH) sensors from Driesen + Kern GmbH were installed at 2 m above ground (Appelhans et al., 2016). The temperature and relative humidity sensors have an accuracy of ±0.5 °C and ±2%, respectively (Appelhans et al., 2016). Data were recorded at 5 minutes interval and aggregated to hourly time interval. Details of sensor and site characteristics are provided in Appelhans et al. (2016).

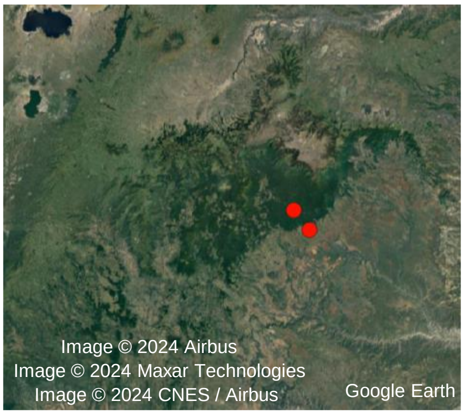

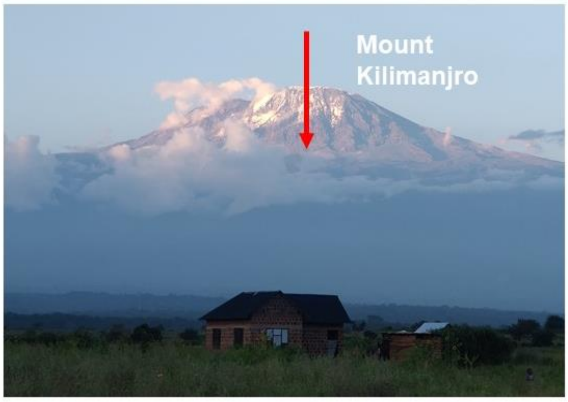

Supplementary Figure 14. Locations of weather stations used for external validation in Kilimanjaro montane cloud forest in Tanzania. The background imagery in the left is from Google Earth (sources: Airbus, Maxar Technologies, and CNES). Photo in the right, Netra Bhandari ©2024.

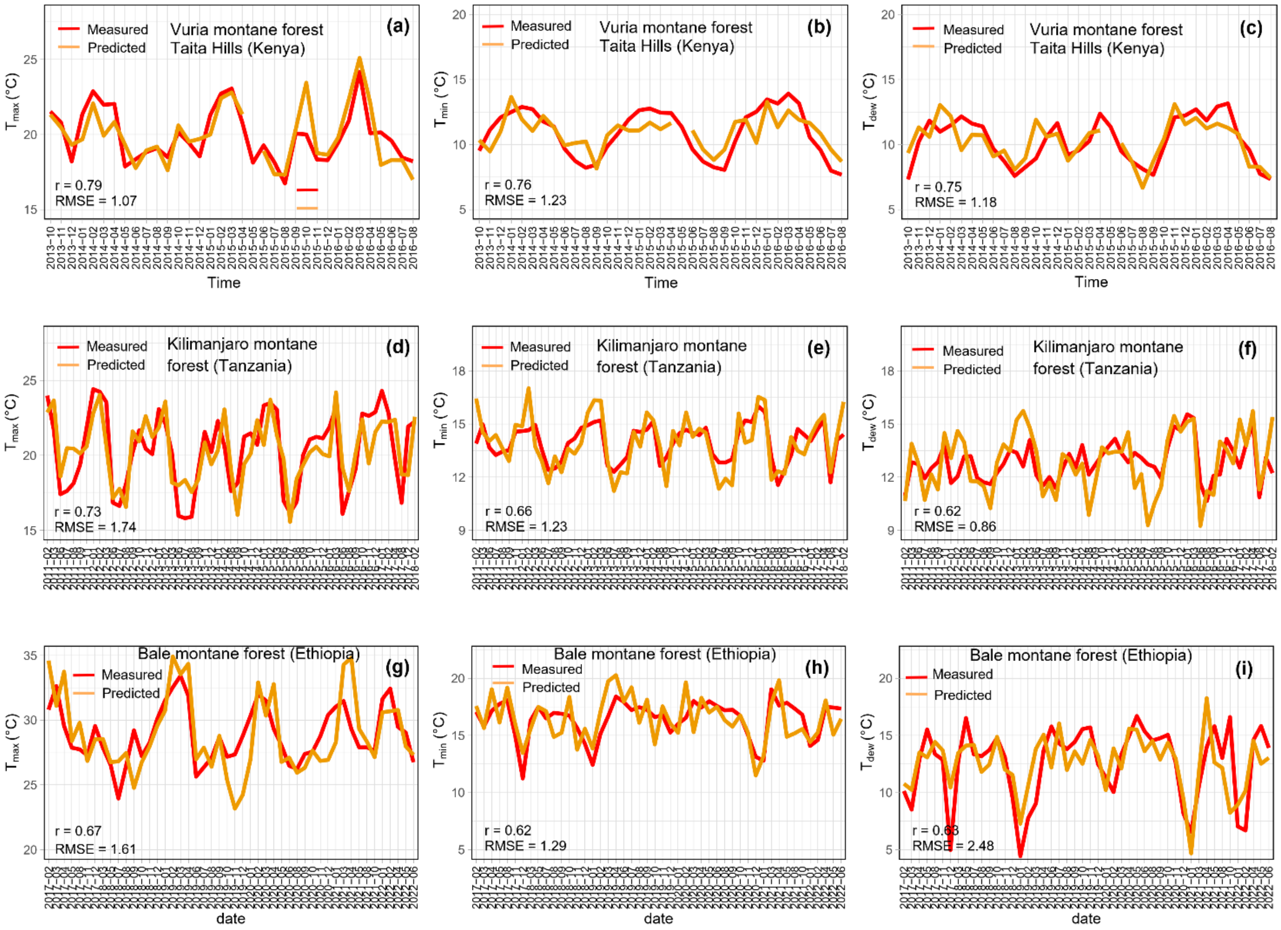

Supplementary Figure 15. Comparison between measured and modeled monthly T_{max} (maximum air temperature), T_{min} (minimum air temperature), and T_{dew} (dew point temperature) using independent external validation weather station data over (a-c) Vuria montane forest in Kenya, (d-f) Kilimanjaro montane forest in Tanzania, and (g-i) Bale montane forest in Ethiopia. Months with > 10% missing data in weather stations in Kilimanjaro and Bale montane forest were excluded. See site description below.

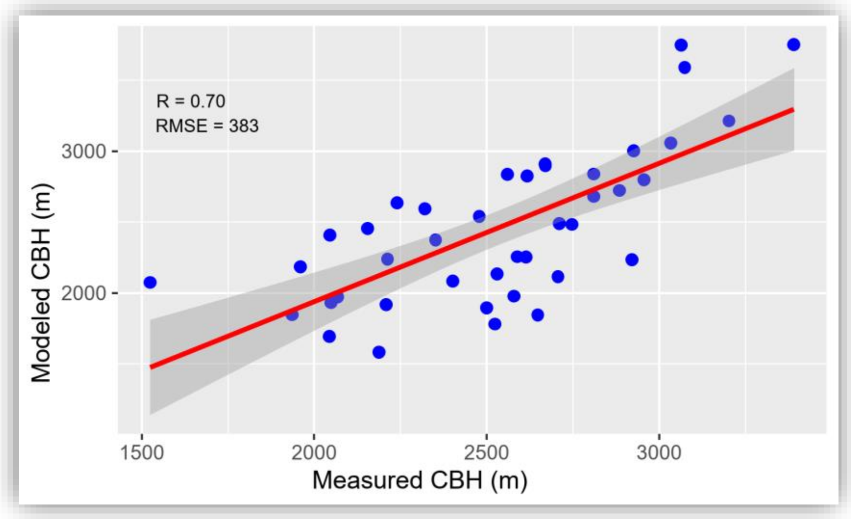

Supplementary Figure 16. Comparison between measured cloud base height (CBH) using Vaisala CT25K Laser Ceilometer and modeled CBH during 2013-2017 at Welgegund measurement site in South Africa (see location of the measurement site in Fig.1 in the main manuscript and site description below).

Welgegund, South Africa

Welgegund atmospheric measurement station (26.570146 °S, 26.939311 °E, 1480 m a.s.l.) is located in a savannah grassland environment approx. 100 km southwest of Johannesburg (Jaars et al., 2016). A Vaisala CT25K ceilometer has been operating at Welgegund station since September 2012, here we utilize five-year period from 2013 to 2017. Vaisala CT25K operates at 15 s temporal and 30 m spatial resolution; height range is 7.5 km. The internal software reports up to three cloud base heights, we used the first (lowest) cloud base for the comparison.

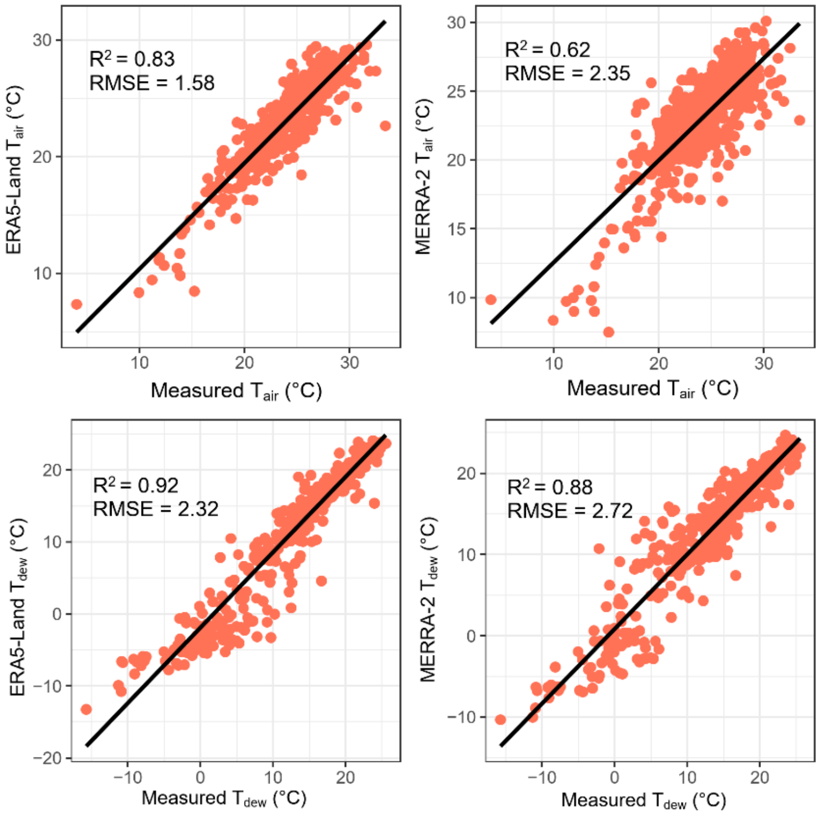

Supplementary Figure 17. Comparison of maximum air temperature (T_{air}) and dewpoint temperature (T_{dew}) from ERA5-Land and MERRA-2 against in situ measurement from GSOD station data.

Supplementary References

1. Global Administrative Areas (GADM) (2015 - 2022). University of California, Berkely. [digital geospatial data]. Available online: http://www.gadm.org [22.07.2024]
2. Hansen, *et al.* High-Resolution Global Maps of 21st-Century Forest Cover Change. *Science*, 342, 850-853 (2013).
3. Katata, G., H. Nagai, T. Wrzesinsky, O. Klemm, W. Eugster, and R. Burkard. Development of a land surface model including cloud water deposition on vegetation, *J. Appl. Meteorol. Climatol.*, 47, 2129–2146 (2008).
4. Potapov, et al. Mapping Global forest Canopy Height through Integration of GEDI and Landsat Data. Remote Sensing Environ. 253, 112165 (2021).
5. Potapov, et al. The Global 2000-2020 Land Cover and Land Use Change Dataset Derived From the Landsat Archive: First Results. Front. Remote Sens. 3 (2022).
6. Myneni et al. MODIS/Terra+Aqua Leaf Area Index/FPAR 4-Day L4 Global 500m SIN Grid V061 [LAI]. NASA EOSDIS Land Processes Distributed Active Archive Center. Accessed 2024-02-24 from https://doi.org/10.5067/MODIS/MCD15A3H.061(2021)
7. Pu et al.. Evaluation of the MODIS LAI/FPAR Algorithm Based on 3D-RTM Simulations: A Case Study of Grassland. *Remote Sens. 12*, 3391(2020).
8. NOAA National Centers of Environmental Information (1999). Global Surface Summary of the Day - GSOD. 1.0. [air temperature and dew point temperature]. NOAA National Centers for Environmental Information. Accessed [05.02.2024]
9. Ploton, P., Mortier, F., Réjou-Méchain, M. *et al.* Spatial validation reveals poor predictive performance of large-scale ecological mapping models. *Nat Commun* 11, 4540 (2020).
10. Räsänen, M., Chung, M., Katurji, M., Pellikka, P., Rinne, J., & Katul, G. G. Similarity in fog and rainfall intermittency. *Geophysical Research Letters*, 45, 10,691–10,699 (2018).
11. Appelhans, T., Mwangomo, E., Otte, I., Detsch, F., Nauss, T. and Hemp, A. Eco-meteorological characteristics of the southern slopes of Kilimanjaro, Tanzania. Int. J. Climatol., 36: 3245-3258 (2016).
12. Jaars, K. et al. Measurements of biogenic volatile organic compounds at a grazed savannah grassland agricultural landscape in South Africa. Atmos. Chem. Phys. 16, 15665–15688 (2016).
13. Olofsson et al. Good practices for estimating area and assessing accuracy of land change Remote Sens. Environ. 148, 42-57 (2014).
14. Abera et al. Towards tree-based systems disturbance monitoring of tropical mosaic landscape using a time series ensemble learning approach,Remote Sensing of Environment, 299,113876 (2023).
